# Supplementary material for: Integration of In Silico and In Vitro Analysis of Gliotoxin Production Reveals a Narrow Range of Producing Fungal Species
Source: J Fungi (Basel). 2022 Mar 31;8(4):361. doi: 10.3390/jof8040361 (PMC9030297; doi:10.3390/jof8040361)
Supplement: Supplementary file 1 [file jof-08-00361-s001.zip › jof-1633909-supplementary.pdf]

Journal of Fungi

MDPI

	GliZ (transcription factor)						
	Locus tag	Accesion	Score	Query Coverage	E value	Identity	
Aspergillus fumigatus Af293	AFUA_6G09630	XP_750852	981	100%	0.0	100.00%	
Aspergillus fumigatus A1163	AFUB_075680	EDP49538	979	100%	0.0	99.80%	
Aspergillus fischerii NRRL 181	NFIA_055320	XP_001258080	679	100%	0.0	77.39%	
Aspergillus lentulus IFM 54703	ALT_0512	GAQ03191	661	100%	0.0	76.36%	
Aspergillus novofumigatus IBT 16806	P174DRAFT_366288	XP_024683986	662	100%	0.0	76.42%	
Aspergillus udagawae IFM 46973	AUD_6969	GAO88009.1	627	99%	0.0	73.93%	
Aspergillus turcosus HMR AF 1038	CFD26_100067	RLL94546	52.8	21%	3,00E-06	33.65%	Outside the cluster
Aspergillus thermomutatus HMR Af 39	CDV56_100588	XP_026610439	497	93%	3,00E-173	61.67%	
Penicillium flavigenum IBT 14082	PENFLA_c002G10567	OQE30980	58.9	13%	4,00E-08	39.71%	Outside the cluster
Penicillium expansum MD-8	PEX2_011700	XP_016603801	63.2	34%	2,00E-09	30.11%	
Bipolaris vicotriae FI3	COCVIDRAFT_21711	XP_014562255	102	14%	2,00E-22	65.28%	
Elsinoe ampelina CECT 20119	BDZ85DRAFT_46612	KAF2219168	50.1	11%	2,00E-05	45.45%	
Coleophoma cylindrospora BP6252	BP6252_11167	RDW63622	59.7	41%	2,00E-08	27.80%	Outside the cluster
Colletotrichum fructicola CGMCC3.17371	CGMCC3_g536	XP_031892997	58.2	15%	1,00E-07	33.78%	Outside the cluster
Colletotrichum asianum ICMP 18580	GQ607_003323	KAF0329374	58.9	15%	5,00E-08	33.33%	Outside the cluster
Trichoderma virens Gv29-8	TRIV-IDRAFT_191964	EHK21730	80.9	13%	4,00E-15	53.03%	Outside the cluster
Torrubiella hemipterigena BCC 1449	VHEMI02390	CEJ82319	57.0	13%	2,00E-07	46.75%	
Penicilliopsis zonata CBS 506.65	ASPZO-DRAFT_26543	XP_022580460	53.9	10%	2,00E-06	47.17%	
Trichoderma reesei RUT C-30	M419DRAFT_91236	ETR97558	54.7	17%	1,00E-06	37.36%	
Rhizodiscina lignyota CBS 133067	NA57DRAFT_32427	KAF2103428	48.5	15%	1,00E-04	33.33%	
Trichoderma reesei QM6a	TRIRE-DRAFT_52368	XP_006969323	54.7	17%	1,00E-06	37.36%	Outside the cluster
Trichoderma harzianum TR274	CI102_1866	PKK53447	48.9	12%	8,00E-05	35.48%	
Trichoderma harzianum CBS 226.95	M431DRAFT_145743	XP_024772674	49.3	12%	7,00E-05	35.48%	
Trichoderma parareesei CBS 125925	A9Z42_0037330	OTA00068	50.8	15%	2,00E-05	37.18%	Outside the cluster

	GliI (Aminotransferase)						
	**Locus tag**	**Accesion**	**Score**	**Query Cover-age**	E value	Identity	
Aspergillus fumigatus Af293	AFUA_6G09640	XP_750853	898	100%	0.0	100.00%	
Aspergillus fumigatus A1163	AFUB_075690	EDP49539	896	100%	0.0	99.77%	
Aspergillus fischerii NRRL 181	NFIA_055330	XP_001258081	825	99%	0.0	91.94%	
Aspergillus lentulus IFM 54703	ALT_0511	GAQ03190	783	99%	0.0	87.79%	
Aspergillus novofumigatus IBT 16806	P174DRAFT_458936	XP_024683985	774	99%	0.0	87.07%	
Aspergillus udagawae IFM 46973	AUD_6970	GAO88010	773	99%	0.0	85.81%	
Aspergillus turcosus HMR AF 1038	CFD26_103418	RLL93875	754	100%	0.0	84.21%	
Aspergillus thermomutatus HMR Af 39	CDV56_100893	RHZ44668	737	97%	0.0	84.15%	
Penicillium flavigenum IBT 14082	PENFLA_c013G03953	OQE22181	521	98%	0.0	60.46%	
Penicillium expansum MD-8	PEX2_011690	XP_016603800	347	90%	6,00E-116	48.85%	
Bipolaris vicotriae FI3	COCVIDRAFT_84446	EUN32660	347	95%	2,00E-114	43.91%	
Elsinoe ampelina CECT 20119	BDZ85DRAFT_206595	KAF2219174	235	89%	4,00E-72	36.27%	
Coleophoma cylindrospora BP6252	BP6252_07497	RDW70934	419	96%	1,00E-142	51.05%	
Colletotrichum fructicola CGMCC3.17371	CGMCC3_g2277	XP_031891505	180	88%	1,00E-50	31.00%	Outside the cluster
Colletotrichum asianum ICMP 18580	GQ607_003912	KAF0328887.1	166	37%	1,00E-48	52.80%	
Trichoderma virens Gv29-8	TRIV-IDRAFT_53497	EHK22001	437	96%	2,00E-150	51.77%	
Torrubiella hemipterigena BCC 1449	VHEMI02396	CEJ82325	416	97%	1,00E-141	48.00%	
Penicilliopsis zonata CBS 506.65	ASPZO-DRAFT_68501	XP_022580458	344	95%	2,00E-114	44.34%	
Trichoderma reesei RUT C-30	M419DRAFT_133449	ETR98471	316	96%	4,00E-103	38.99%	
Rhizodiscina lignyota CBS 133067	NA57DRAFT_49544	KAF2092843	326	96%	1,00E-106	41.28%	
Trichoderma reesei QM6a	TRIRE-DRAFT_54160	XP_006961383	317	97%	2,00E-103	39.04%	
Trichoderma harzianum TR274	CI102_1859	PKK53438	335	96%	3,00E-110	40.60%	
Trichoderma harzianum CBS 226.95	M431DRAFT_555938	XP_024772667	335	96%	1,00E-110	40.60%	
Trichoderma parareesei CBS 125925	A9Z42_0047280	OTA04145	101	22%	8,00E-25	44.44%	

	GliJ (dipeptidase)						
	Locus tag	Accesion	Score	Query Coverage	E value	Identity	
Aspergillus fumigatus Af293	AFUA_6G09650	XP_750854	796	100%	0.0	100.00%	
Aspergillus fumigatus A1163	AFUB_075700	EDP49540	794	100%	0.0	99.74%	
Aspergillus fischerii NRRL 181	NFIA_055340	XP_001258082	754	100%	0.0	95.36%	
Aspergillus lentulus IFM 54703	ALT_0510	GAQ03189	734	99%	0.0	91.47%	
Aspergillus novofumigatus IBT 16806	P174DRAFT_367031	XP_024683984	726	100%	0.0	91.24%	
Aspergillus udagawae IFM 46973	AUD_6971	GAO88011	651	100%	0.0	83.51%	
Aspergillus turcosus HMR AF 1038	CFD26_103275	RLL93874	712	98%	0.0	89.06%	
Aspergillus thermomutatus HMR Af 39	XP_026610441	CDV56_101176	712	100%	0.0	88.40%	
Penicillium flavigenum IBT 14082	PENFLA_c013G03435	OQE22014	435	97%	7,00E-152	57.74%	
Penicillium expansum MD-8	PEX2_011790	KGO63390.1	248	97%	1,00E-78	39.58%	
Bipolaris vicotriae FI3	COCVIDRAFT_84229	EUN32653	439	94%	7,00E-153	58.54%	
Elsinoe ampelina CECT 20119	BDZ85DRAFT_305117	KAF2219171	399	92%	3,00E-137	54.42%	
Coleophoma cylindrospora BP6252	BP6252_07501	RDW70938	409	96%	1,00E-140	53.28%	
Colletotrichum fructicola CGMCC3.17371	CGMCC3_g11502	XP_031881996	390	93%	2,00E-133	52.69%	
Colletotrichum asianum ICMP 18580	GQ607_001290	KAF0331544	295	93%	3,00E-95	41.96%	Outside the cluster
Trichoderma virens Gv29-8	TRIV-IDRAFT_216163	EHK21959	419	99%	1,00E-144	52.81%	
Torrubiella hemipterigena BCC 1449	VHEMI02395	CEJ82324	423	96%	2,00E-146	55.29%	
Penicilliopsis zonata CBS 506.65	ASPZO-DRAFT_547278	OJJ45949	420	97%	2,00E-145	54.83%	
Trichoderma reesei RUT C-30	M419DRAFT_133453	ETR98475.1	405	96%	3,00E-139	54.52%	
Rhizodiscina lignyota CBS 133067	NA57DRAFT_49457	KAF2092846	390	95%	2,00E-133	51.60%	
Trichoderma reesei QM6a	TRIRE-DRAFT_103119	XP_006961835	405	95%	3,00E-139	54.52%	
Trichoderma harzianum TR274	CI102_1863	PKK53444	369	90%	1,00E-125	51.97%	
Trichoderma harzianum CBS 226.95	M431DRAFT_555948	PTB52994	367	90%	5,00E-125	51.69%	
Trichoderma parareesei CBS 125925	A9Z42_0047000	OTA04141	410	95%	2,00E-141	54.28%	

	GliP (Nonribosomal peptide synthase)					
	**Locus tag**	**Accesion**	**Score**	**Query Coverage**	**E value**	**Identity**
Aspergillus fumigatus Af293	AFUA_6G09660	EAL88817	4396	100%	0.0	100.00%
Aspergillus fumigatus A1163	AFUB_075710	EDP49541	4386	100%	0.0	99.77%
Aspergillus fischerii NRRL 181	NFIA_055350	XP_001258083	4152	100%	0.0	94.75%
Aspergillus lentulus IFM 54703	ALT_0509	GAQ03188	4101	100%	0.0	93.13%
Aspergillus novofumigatus IBT 16806	P174DRAFT_511160	XP_024683983	4012	100%	0.0	92.06%
Aspergillus udagawae IFM 46973	AUD_6972	GAO88012	3920	100%	0.0	89.30%
Aspergillus turcosus HMR AF 1038	CFD26_102651	RLL93873	3896	100%	0.0	88.42%
Aspergillus thermomutatus HMR Af 39	CDV56_101444	XP_026610442	3779	100%	0.0	87.07%
Penicillium flavigenum IBT 14082	PENFLA_c013G03821	OQE22222	2652	99%	0.0	60.95%
Penicillium expansum MD-8	PEX2_011780	XP_016603809	2644	99%	0.0	61.24%
Bipolaris vicotriae FI3	COCVIDRAFT_32734	EUN32662	1969	95%	0.0	47.29%
Elsinoe ampelina CECT 20119	BDZ85DRAFT_305103	KAF2219165	1712	99%	0.0	44.93%
Coleophoma cylindrospora BP6252	BP6252_07505	RDW70942	1642	95%	0.0	44.21%
Colletotrichum fructicola CGMCC3.17371	CGMCC3_g11493	XP_031882093	1640	94%	0.0	44.00%
Colletotrichum asianum ICMP 18580	GQ607_003908	KAF0328883	1627	94%	0.0	44.09%
Trichoderma virens Gv29-8	TRIV-IDRAFT_78708	EHK22005	1621	94%	0.0	43.99%
Torrubiella hemipterigena BCC 1449	VHEMI02393	CEJ82322	1718	94%	0.0	41.43%
Penicilliopsis zonata CBS 506.65	ASPZO-DRAFT_160119	XP_022580453	1481	96%	0.0	42.54%
Trichoderma reesei RUT C-30	M419DRAFT_133451	ETR98473	1461	98%	0.0	39.94%
Rhizodiscina lignyota CBS 133067	NA57DRAFT_81988	KAF2092841	1461	98%	0.0	39.34%
Trichoderma reesei QM6a	TRIRE-DRAFT_24586	XP_006961011	1461	98%	0.0	39.94%
Trichoderma harzianum TR274	CI102_1861	PKK53446	1453	99%	0.0	38.55%
Trichoderma harzianum CBS 226.95	M431DRAFT_496332	XP_024772669	1453	99%	0.0	38.55%
Trichoderma parareesei CBS 125925	A9Z42_0047170	OTA04143	1433	99%	0.0	39.13%

	GliC (P450 oxidorreductase)					
	**Locus tag**	**Accesion**	**Score**	**Query Coverage**	E value	Identity
Aspergillus fumigatus Af293	AFUA_6G09670	EAL88818	1060	100%	0.0	100.00%
Aspergillus fumigatus A1163	AFUB_075720	EDP49542	1056	100%	0.0	99.61%
Aspergillus fischerii NRRL 181	NFIA_055360	XP_001258084	1019	100%	0.0	95.71%
Aspergillus lentulus IFM 54703	ALT_0508	GAQ03187	1003	100%	0.0	92.98%
Aspergillus novofumigatus IBT 16806	P174DRAFT_481241	XP_024683982	982	100%	0.0	92.59%
Aspergillus udagawae IFM 46973	AUD_6973	GAO88013	973	99%	0.0	90.96%
Aspergillus turcosus HMR AF 1038	CFD26_102418	RLL93872	930	100%	0.0	87.16%
Aspergillus thermomutatus HMR Af 39	CDV56_102362	XP_026610443	948	99%	0.0	89.00%
Penicillium flavigenum IBT 14082	PENFLA_c013G08698	OQE22156	704	99%	0.0	64.90%
Penicillium expansum MD-8	PEX2_011770	XP_016603808	536	77%	0.0	64.06%
Bipolaris vicotriae FI3	COCVIDRAFT_21720	EUN32661	524	89%	0.0	54.72%
Elsinoe ampelina CECT 20119	BDZ85DRAFT_305107	KAF2219166	486	86%	1,00E-168	54.75%
Coleophoma cylindrospora BP6252	BP6252_07504	RDW70941	318	62%	3,00E-104	49.54%
Colletotrichum fructicola CGMCC3.17371	CGMCC3_g11494	KAE9572544	296	69%	8,00E-95	43.96%
Colletotrichum asianum ICMP 18580	GQ607_003909	KAF0328884	360	78%	7,00E-119	45.28%
Trichoderma virens Gv29-8	TRIV-IDRAFT_216161	EHK22004	446	90%	6,00E-152	47.64%
Torrubiella hemipterigena BCC 1449	VHEMI02392	CEJ82321	434	90%	6,00E-147	46.67%
Penicilliopsis zonata CBS 506.65	ASPZO-DRAFT_167643	OJJ45944	425	94%	4,00E-144	44.65%
Trichoderma reesei RUT C-30	M419DRAFT_133450	ETR98472	443	96%	1,00E-150	46.28%
Rhizodiscina lignyota CBS 133067	NA57DRAFT_49506	KAF2092842	422	88%	3,00E-143	45.43%
Trichoderma reesei QM6a	TRIRE-DRAFT_53168	EGR53014	441	89%	8,00E-151	48.59%
Trichoderma harzianum TR274	CI102_1860	PKK53445	390	82%	1,00E-130	48.46%
Trichoderma harzianum CBS 226.95	M431DRAFT_521249	XP_024772668	407	89%	6,00E-137	47.38%
Trichoderma parareesei CBS 125925	A9Z42_0046970	OTA04144	437	87%	1,00E-148	49.11%

	GliM (O-methyltransferase)						
	**Locus tag**	**Accesion**	**Score**	**Query Coverage**	E value	Identity	
Aspergillus fumigatus Af293	AFUA_6G09680	EAL88819	887	100%	0.0	100.00%	
Aspergillus fumigatus A1163	AFUB_075730	EDP49543	887	100%	0.0	100.00%	
Aspergillus fischerii NRRL 181	NFIA_055370	EAW16188	854	100%	0.0	96.06%	
Aspergillus lentulus IFM 54703	ALT_0507	GAQ03186	840	100%	0.0	93.97%	
Aspergillus novofumigatus IBT 16806	P174DRAFT_368520	XP_024683981	840	100%	0.0	93.50%	
Aspergillus udagawae IFM 46973	AUD_6974	GAO88014	826	100%	0.0	92.58%	
Aspergillus turcosus HMR AF 1038	CFD26_102266	RLL93871	832	99%	0.0	92.09%	
Aspergillus thermomutatus HMR Af 39	CDV56_102666	RHZ44672	758	99%	0.0	83.48%	
Penicillium flavigenum IBT 14082	PENFLA_c013G07948	OQE22128	657	100%	0.0	71.82%	
Penicillium expansum MD-8	PEX2_011760	KGO63387	653	95%	0.0	73.67%	
Bipolaris vicotriae FI3	COCVIDRAFT_84476	EUN32658	437	95%	7E-151	55.66%	
Elsinoe ampelina CECT 20119	BDZ85DRAFT_285636	KAF2219170	442	93%	5E-153	50.86%	
Coleophoma cylindrospora BP6252	BP6252_07506	RDW70943	470	96%	6E-164	52.40%	
Colletotrichum fructicola CGMCC3.17371	CGMCC3_g11498	KAE9572548	459	93%	2E-159	51.84%	
Colletotrichum asianum ICMP 18580	GQ607_014591	KAF0318223	311	97%	1E-96	41.78%	
Trichoderma virens Gv29-8	TRIV-IDRAFT_216154	EHK21998	483	96%	4E-169	55.74%	
Torrubiella hemipterigena BCC 1449	VHEMI02391	CEJ82320	488	93%	5E-171	58.31%	
Penicilliopsis zonata CBS 506.65	ASPZO-DRAFT_68902	OJJ45945	488	94%	3E-171	54.90%	
Trichoderma reesei RUT C-30	M419DRAFT_38490	ETR98480	350	85%	8E-117	46.92%	
Rhizodiscina lignyota CBS 133067	NA57DRAFT_49558	KAF2092854	354	93%	1,00E-118	42.96%	Outside the cluster
Trichoderma reesei QM6a	TRIRE-DRAFT_53025	EGR53018	350	85%	8,00E-117	46.92%	
Trichoderma harzianum TR274	CI102_1869	PKK53418	347	87%	1,00E-115	44.88%	
Trichoderma harzianum CBS 226.95	M431DRAFT_145749	PTB52999	347	87%	1,00E-115	44.88%	
Trichoderma parareesei CBS 125925	A9Z42_0047250	OTA04135	339	77%	2,00E-113	48.22%	

	GliG (glutathione S-transferase)						
	**Locus tag**	**Accesion**	**Score**	**Query Coverage**	E value	Identity	
Aspergillus fumigatus Af293	AFUA_6G09690	EAL88820	494	100%	1,00E-179	100.00%	
Aspergillus fumigatus A1163	AFUB_075740	EDP49544	494	100%	3,00E-179	99.58%	
Aspergillus fischerii NRRL 181	NFIA_055380	EAW16189	488	100%	1,00E-176	97.92%	
Aspergillus lentulus IFM 54703	ALT_0506	GAQ03185	481	100%	4,00E-174	95.83%	
Aspergillus novofumigatus IBT 16806	P174DRAFT_458931	PKX95385	479	100%	1,00E-173	96.25%	
Aspergillus udagawae IFM 46973	AUD_6975	GAO88015	481	100%	3,00E-174	95.83%	
Aspergillus turcosus HMR AF 1038	CFD26_102131	RLL93870	463	100%	4,00E-167	91.67%	
Aspergillus thermomutatus HMR Af 39	CDV56_102892	RHZ44673	466	100%	4,00E-168	92.08%	
Penicillium flavigenum IBT 14082	PENFLA_c013G05520	OQE22140	408	98%	3,00E-145	81.78%	
Penicillium expansum MD-8	PEX2_011750	KGO63386	400	98%	2,00E-142	80.08%	
Bipolaris vicotriae FI3	COCVIDRAFT_32732	EUN32657	345	97%	3,00E-120	70.39%	
Elsinoe ampelina CECT 20119	BDZ85DRAFT_46730	KAF2219179	218	91%	5,00E-70	44.00%	
Coleophoma cylindrospora BP6252	BP6252_07498	RDW70935	300	95%	2,00E-102	58.89%	
Colletotrichum fructicola CGMCC3.17371	CGMCC3_g11496	KAE9572543	211	60%	6,00E-69	69.66%	
Colletotrichum asianum ICMP 18580	GQ607_002621	KAF0330291	76.3	80%	1,00E-15	28.37%	Outside the cluster
Trichoderma virens Gv29-8	TRIVIDRAFT_216157	EHK22000	313	95%	5,00E-108	68.12%	
Torrubiella hemipterigena BCC 1449	VHEMI02397	CEJ82326	312	95%	1,00E-107	64.78%	
Penicilliopsis zonata CBS 506.65	ASPZODRAFT_68717	OJJ45947	178	86%	1,00E-54	45.50%	
Trichoderma reesei RUT C-30	M419DRAFT_64348	ETR98476	164	82%	1,00E-49	43.07%	
Rhizodiscina lignyota CBS 133067	NA57DRAFT_69594	KAF2092847	170	86%	1,00E-51	42.45%	
Trichoderma reesei QM6a	TRIREDRAFT_32402	EGR53016	164	82%	1,00E-49	43.07%	
Trichoderma harzianum TR274	CI102_1864	PKK53449	156	82%	4,00E-46	40.59%	
Trichoderma harzianum CBS 226.95	M431DRAFT_496335	PTB52995	156	82%	4,00E-46	40.59%	
Trichoderma parareesei CBS 125925	A9Z42_0047200	OTA04140	171	90%	7,00E-52	41.82%	

	**GliK (Gamma-glutamylcyclotransferase)**						
	**Locus tag**	**Accesion**	**Score**	**Query Coverage**	E value	Identity	
Aspergillus fumigatus Af293	AFUA_6G09700	EAL88821	560	100%	0.0	100.00%	
Aspergillus fumigatus A1163	AFUB_075750	EDP49545	560	100%	0.0	100.00%	
Aspergillus fischerii NRRL 181	NFIA_055390	EAW16190.1	511	98%	0.0	92.19%	
Aspergillus lentulus IFM 54703	ALT_0505	GAQ03184	508	98%	0.0	90.71%	
Aspergillus novofumigatus IBT 16806	P174DRAFT_429762	PKX95384	504	98%	0.0	90.33%	
Aspergillus udagawae IFM 46973	AUD_6976	GAO88016	477	98%	2,00E-171	88.48%	
Aspergillus turcosus HMR AF 1038	CFD26_102022	RLL93869	492	96%	2,00E-177	88.64%	
Aspergillus thermomutatus HMR Af 39	CDV56_103027	RHZ44674	483	96%	7,00E-174	86.36%	
Penicillium flavigenum IBT 14082	PENFLA_c013G07766	OQE22030	199	53%	3,00E-63	68.03%	
Penicillium expansum MD-8	PEX2_011740	KGO63385	196	53%	5,00E-62	66.67%	
Bipolaris vicotriae FI3	COCVIDRAFT_84291	EUN32656.1	259	92%	1,00E-85	55.04%	
Elsinoe ampelina CECT 20119	BDZ85DRAFT_285643	KAF2219178	190	91%	2,00E-58	40.08%	
Coleophoma cylindrospora BP6252	BP6252_07500	RDW70937	235	89%	5,00E-76	46.12%	
Colletotrichum fructicola CGMCC3.17371	CGMCC3_g9322	KAE9574802	65.9	79%	4,00E-11	25.09%	Outside the cluster
Colletotrichum asianum ICMP 18580	GQ607_003911	KAF0328886	221	90%	8,00E-71	44.13%	
Trichoderma virens Gv29-8	TRIV-IDRAFT_151379	EHK22002	239	89%	4,00E-78	46.94%	
Torrubiella hemipterigena BCC 1449	VHEMI02394	CEJ82323	254	93%	1,00E-83	47.91%	
Penicilliopsis zonata CBS 506.65	ASPZO-DRAFT_17375	OJJ45946	201	91%	1,00E-62	43.18%	
Trichoderma reesei RUT C-30	M419DRAFT_88682	ETR98474	197	94%	4,00E-61	39.69%	
Rhizodiscina lignyota CBS 133067	NA57DRAFT_69592	KAF2092845	208	91%	2,00E-65	42.97%	
Trichoderma reesei QM6a	TRIRE-DRAFT_54784	EGR52475	197	94%	4,00E-61	39.69%	
Trichoderma harzianum TR274	CI102_1862	PKK53443	197	94%	3,00E-61	40.93%	
Trichoderma harzianum CBS 226.95	M431DRAFT_88512	PTB5299	197	94%	3,00E-61	40.93%	
Trichoderma parareesei CBS 125925	A9Z42_0047010	OTA04142	197	94%	6,00E-61	39.30%	

	GliA (MFS - efflux channel)						
	**Locus tag**	**Accesion**	**Score**	**Query Coverage**	E value	Identity	
Aspergillus fumigatus Af293	AFUA_6G09710	EAL88822	1081	100%	0.0	100.00%	
Aspergillus fumigatus A1163	AFUB_075760	EDP49546	1081	100%	0.0	100.00%	
Aspergillus fischerii NRRL 181	NFIA_055400	EAW16191	1040	99%	0.0	96.30%	
Aspergillus lentulus IFM 54703	ALT_0504	GAQ03183	969	100%	0.0	93.91%	
Aspergillus novofumigatus IBT 16806	P174DRAFT_367489	PKX95383	978	100%	0.0	94.10%	
Aspergillus udagawae IFM 46973	AUD_6977	GAO88017	996	100%	0.0	92.80%	
Aspergillus turcosus HMR AF 1038	CFD26_101781	RLL93868	946	99%	0.0	89.13%	
Aspergillus thermomutatus HMR Af 39	CDV56_103194	RHZ44675	926	100%	0.0	89.30%	
Penicillium flavigenum IBT 14082	PENFLA_c008G02142	OQE25695	451	98%	1,00E-151	42.49%	Outside the cluster
Penicillium expansum MD-8	PEX2_081320	KGO61278	501	97%	2,00E-170	46.75%	Outside the cluster
Bipolaris vicotriae FI3	COCVIDRAFT_108412	EUN23555	469	96%	5,00E-160	44.28%	Outside the cluster
Elsinoe ampelina CECT 20119	BDZ85DRAFT_172137	KAF2221582	512	91%	2,00E-177	51.51%	Outside the cluster
Coleophoma cylindrospora BP6252	BP6252_07509	RDW70946	376	98%	5,00E-123	35.99%	
Colletotrichum fructicola CGMCC3.17371	CGMCC3_g11500	KAE9572451	457	90%	3,00E-155	45.64%	
Colletotrichum asianum ICMP 18580	GQ607_000787	KAF0331667	527	97%	0.0	48.86%	Outside the cluster
Trichoderma virens Gv29-8	TRIV-IDRAFT_216146	EHK22068	449	88%	7,00E-152	45.42%	Outside the cluster
Torrubiella hemipterigena BCC 1449	VHEMI01223	CEJ81073	452	97%	7,00E-153	43.56%	Outside the cluster
Penicilliopsis zonata CBS 506.65	ASPZO-DRAFT_133807	OJJ45940	494	88%	2,00E-170	53.00%	
Trichoderma reesei RUT C-30	M419DRAFT_67631	ETS06688	434	97%	3,00E-145	43.75%	Outside the cluster
Rhizodiscina lignyota CBS 133067	NA57DRAFT_42743	KAF2096360	521	96%	4,00E-180	49.33%	Outside the cluster
Trichoderma reesei QM6a	TRIRE-DRAFT_53503	EGR52888	449	97%	1,00E-151	45.18%	Outside the cluster
Trichoderma harzianum TR274	CI102_8510	PKK49097	405	83%	2,00E-135	44.40%	Outside the cluster
Trichoderma harzianum CBS 226.95	M431DRAFT_112430	PTB56957	474	98%	2,00E-161	45.97%	Outside the cluster
Trichoderma parareesei CBS 125925	A9Z42_0045760	OTA04036	446	97%	3,00E-150	45.57%	Outside the cluster

	GliN (methyltransferase)						
	Locus tag	Accesion	Score	Query Coverage	E value	Identity	
Aspergillus fumigatus Af293	AFUA_6G09720	EAL88823	580	100%	0.0	100.00%	
Aspergillus fumigatus A1163	AFUB_075770	EDP49547	580	100%	0.0	100.00%	
Aspergillus fischerii NRRL 181	NFIA_055410	EAW16192	559	100%	0.0	94.68%	
Aspergillus lentulus IFM 54703	ALT_0503	GAQ03182	548	100%	0.0	92.55%	
Aspergillus novofumigatus IBT 16806	P174DRAFT_502743	PKX95382	471	100%	5,00E-169	84.04%	
Aspergillus udagawae IFM 46973	AUD_6978	GAO88018	560	100%	0.0	95.74%	
Aspergillus turcosus HMR AF 1038	CFD26_101626	RLL93867	526	100%	0.0	90.49%	
Aspergillus thermomutatus HMR Af 39	CDV56_103425	RHZ44676	521	100%	0.0	86.88%	
Penicillium flavigenum IBT 14082	PENFLA_c013G04011	OQE22012	138	96%	5E-38	32.87%	
Penicillium expansum MD-8	PEX2_011710	KGO63382	137	96%	8,00E-38	32.87%	
Bipolaris vicotriae FI3	COCVIDRAFT_11169	EUN32655	128	98%	2,00E-34	30.88%	
Elsinoe ampelina CECT 20119	BDZ85DRAFT_46687	KAF2219173	110	96%	2,00E-27	27.90%	
Coleophoma cylindrospora BP6252	BP6252_07499	RDW70936	341	97%	3,00E-117	55.94%	
Colletotrichum fructicola CGMCC3.17371	CGMCC3_g11495	KAE9572551	333	98%	3,00E-114	56.38%	
Colletotrichum asianum ICMP 18580	GQ607_003910	KAF0328885	339	98%	9,00E-117	57.09%	
Trichoderma virens Gv29-8	TRIVIDRAFT_91355	EHK22003	342	98%	6,00E-118	57.55%	
Torrubiella hemipterigena BCC 1449	VHEMI02398	CEJ82327	132	96%	1,00E-35	28.36%	
Penicilliopsis zonata CBS 506.65	ASPZODRAFT_66834	OJJ46766	77.8	75%	3,00E-15	27.75%	Outside the cluster
Trichoderma reesei RUT C-30	M419DRAFT_11794	ETR98470	108	97%	6,00E-27	27.24%	Outside the cluster
Rhizodiscina lignyota CBS 133067	NA57DRAFT_69591	KAF2092844	118	96%	1,00E-30	29.71%	
Trichoderma reesei QM6a	TRIREDRAFT_103114	EGR53013	108	97%	6,00E-27	27.24%	Outside the cluster
Trichoderma harzianum TR274	CI102_1858	PKK53439	117	97%	3,00E-30	27.14%	
Trichoderma harzianum CBS 226.95	M431DRAFT_118424	PTB52989	117	97%	3,00E-30	27.14%	
Trichoderma parareesei CBS 125925	A9Z42_0047230	OTA04146	105	81%	4,00E-26	29.49%	

	GliF (cytochrome P450 oxidoreductase)						
	Locus tag	Accesion	Score	Query Coverage	E value	Identity	
Aspergillus fumigatus Af293	AFUA_6G09730	EAL88824	1033	100%	0.0	1	
Aspergillus fumigatus A1163	AFUB_075780	EDP49548	1033	100%	0.0	99.80 %	
Aspergillus fischerii NRRL 181	NFIA_055420	EAW16193	1008	100%	0.0	96.63 %	
Aspergillus lentulus IFM 54703	ALT_0502	GAQ03181	997	99%	0.0	95.43 %	
Aspergillus novofumigatus IBT 16806	P174DRAFT_458927	PKX95381	993	100%	0.0	95.44 %	
Aspergillus udagawae IFM 46973	AUD_6979	GAO88019	978	99%	0.0	93.64 %	
Aspergillus turcosus HMR AF 1038	CFD26_101410	RLL93866	948	98%	0.0	92.96 %	
Aspergillus thermomutatus HMR Af 39	CDV56_103568	RHZ44677	938	100%	0.0	90.30 %	
Penicillium flavigenum IBT 14082	PENFLA_c008G02011	PENFLA_c008G02011	182	80%	2,00E-50	31.16 %	Outside the cluster
Penicillium expansum MD-8	PEX2_090260	KGO54773	192	89%	6,00E-54	30.94 %	Outside the cluster
Bipolaris vicotriae FI3	COCVIDRAFT_32765	EUN32704	180	83%	1,00E-49	30.21 %	Outside the cluster
Elsinoe ampelina CECT 20119	BDZ85DRAFT_305131	KAF2219176	551	92%	0.0	58.67 %	
Coleophoma cylindrospora BP6252	BP6252_07507	RDW70944	627	96%	0.0	63.60 %	
Colletotrichum fructicola CGMCC3.17371	CGMCC3_g11497	KAE9572550	517	95%	1,00E-180	56.22 %	
Colletotrichum asianum ICMP 18580	GQ607_003389	KAF0329440	239	99%	7,00E-72	32.95 %	Outside the cluster
Trichoderma virens Gv29-8	TRIVIDRAFT_91346	EHK21999	625	99%	0.0	61.46 %	
Torrubiella hemipterigena BCC 1449	VHEMI06866	CEJ91133	181	78%	8,00E-51	29.46 %	Outside the cluster
Penicilliopsis zonata CBS 506.65	ASPZODRAFT_1360433	OJJ48948	167	88%	2,00E-44	26.54 %	Outside the cluster
Trichoderma reesei RUT C-30	M419DRAFT_85856	ETR99471	205	97%	1,00E-58	30.63 %	Outside the cluster
Rhizodiscina lignyota CBS 133067	NA57DRAFT_52344	KAF2102792	148	66%	2,00E-39	29.82 %	Outside the cluster
Trichoderma reesei QM6a	TRIREDRAFT_66453	EGR46455	205	97%	1,00E-58	30.63 %	Outside the cluster
Trichoderma harzianum TR274	CI102_12316	PKK44048	203	92%	4,00E-58	29.50 %	Outside the cluster
Trichoderma harzianum CBS 226.95	M431DRAFT_21965	PTB47478	218	97%	2,00E-63	29.08 %	Outside the cluster
Trichoderma parareesei CBS 125925	A9Z42_0078160	OTA07011	205	97%	1,00E-58	30.63 %	Outside the cluster

	GliT (disulfide oxidoreductase)						
	**Locus tag**	**Accesion**	**Score**	**Query Coverage**	E value	Identity	
Aspergillus fumigatus Af293	AFUA_6G09740	EAL88825	689	100%	0.0	100.00%	
Aspergillus fumigatus A1163	AFUB_075790	EDP49549	689	100%	0.0	100.00%	
Aspergillus fischerii NRRL 181	NFIA_055430	EAW16194	657	100%	0.0	94.61%	
Aspergillus lentulus IFM 54703	ALT_0501	GAQ03180	637	100%	0.0	91.62%	
Aspergillus novofumigatus IBT 16806	P174DRAFT_511158	PKX95380	637	100%	0.0	91.92%	
Aspergillus udagawae IFM 46973	AUD_6980	GAO88020	649	100%	0.0	93.73%	
Aspergillus turcosus HMR AF 1038	CFD26_101248	RLL93865	630	100%	0.0	90.42%	
Aspergillus thermomutatus HMR Af 39	CDV56_103762	RHZ44678	612	100%	0.0	88.66%	
Penicillium flavigenum IBT 14082	PENFLA_c013G09054	OQE22215	187	92%	1,00E-55	39.56%	
Penicillium expansum MD-8	PEX2_011730	KGO63384	187	92%	1,00E-55	39.56%	
Bipolaris vicotriae FI3	COCVIDRAFT_11166	EUN32652	213	94%	9,00E-66	39.51%	
Elsinoe ampelina CECT 20119	BDZ85DRAFT_276447	KAF2219167	189	94%	2,00E-56	40.25%	
Coleophoma cylindrospora BP6252	BP6252_07502	RDW70939	343	93%	8,00E-117	57.96%	
Colletotrichum fructicola CGMCC3.17371	CGMCC3_g11499	KAE9572552	333	93%	7,00E-113	52.72%	
Colletotrichum asianum ICMP 18580	GQ607_015030	KAF0317720	315	96%	5,00E-106	49.23%	Outside the cluster
Trichoderma virens Gv29-8	TRIVIDRAFT_138628	EHK22124	327	86%	1,00E-110	58.08%	Outside the cluster
Torrubiella hemipterigena BCC 1449	VHEMI02400	CEJ82329	193	92%	4,00E-58	39.94%	
Penicilliopsis zonata CBS 506.65	ASPZODRAFT_68844	OJJ45951	215	87%	8,00E-67	40.14%	
Trichoderma reesei RUT C-30	M419DRAFT_103366	ETR98482	169	92%	1,00E-48	35.85%	Outside the cluster
Rhizodiscina lignyota CBS 133067	NA57DRAFT_49625	KAF2102377	190	92%	9,00E-57	38.46%	Outside the cluster
Trichoderma reesei QM6a	TRIREDRAFT_74278	EGR52478	169	92%	1,00E-48	35.85%	Outside the cluster
Trichoderma harzianum TR274	CI102_1871	PKK53428	159	93%	6,00E-45	34.57%	Outside the cluster
Trichoderma harzianum CBS 226.95	M431DRAFT_88869	PTB53001	159	93%	6,00E-45	34.57%	
Trichoderma parareesei CBS 125925	A9Z42_0047150	OTA04133	171	92%	3,00E-49	35.85%	

Table S1. Contains the best BLAST results for *A. fumigatus* Af293 GliZ, GliI, GliJ, GliP, GliC, GliM, GliG, GliK, GliA, GliN, GliF and GliT proteins. Entries are sorted by score although query coverage, identity and e-value are also shown.

A)

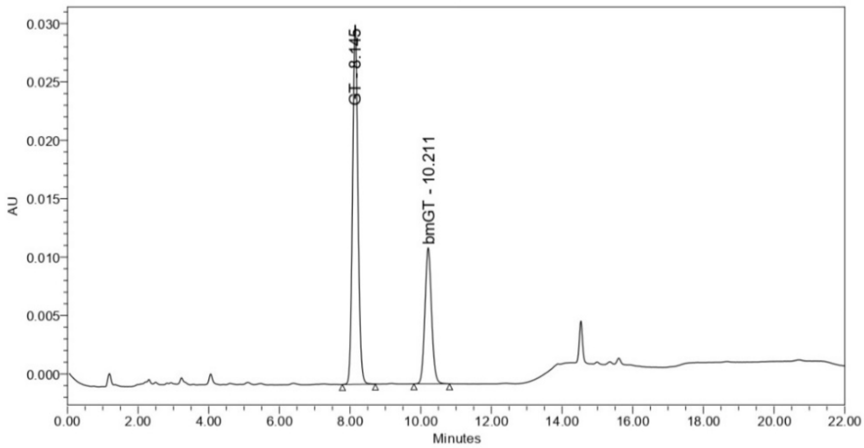

	Peak Name	RT	Area	% Area	Height	Amount	Units
1	GT	8.145	328372	68.17	30792	35.000	ug/ml
2	bmGT	10.211	153324	31.83	11641	35.000	ug/ml

B)

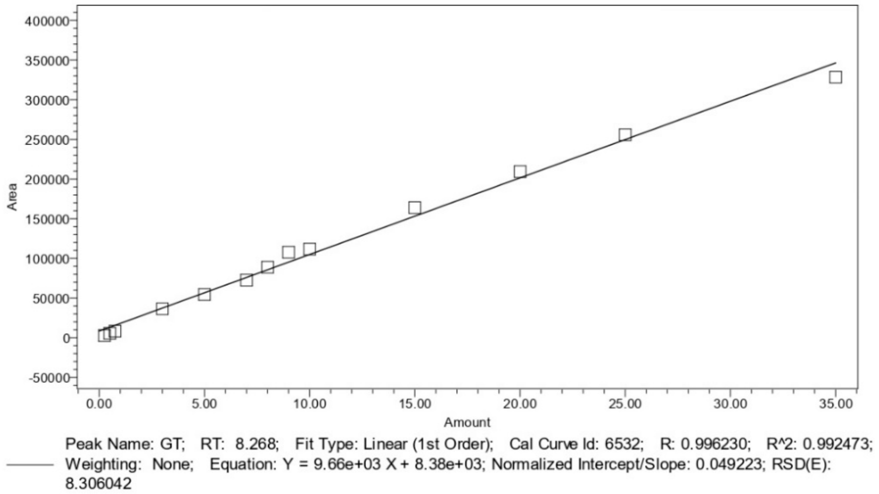

C)

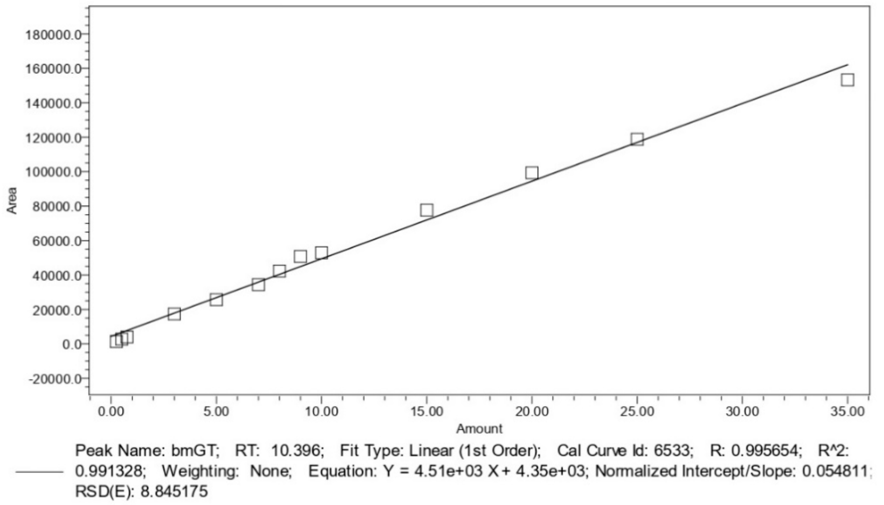

Figure S1. A) HPLC chromatogram of GT and bmGT standard. B) GT curve calibration. C) bmGT curve calibration.
